# Treatment of Laryngeal Verrucous Carcinoma: 28‐Year Retrospective Cohort Study and Literature Review

**DOI:** 10.1002/oto2.50

**Published:** 2023-06-01

**Authors:** Ameen Amanian, Donald W. Anderson, James Scott Durham, Eitan Prisman, Tony Ng, Amanda Hu

**Affiliations:** ^1^ Division of Otolaryngology–Head and Neck Surgery, Department of Surgery University of British Columbia Vancouver Canada; ^2^ Department of Pathology and Laboratory Medicine University of British Columbia Vancouver Canada

**Keywords:** Ackerman's tumor, laryngeal cancer, laryngeal surgery, laryngeal verrucous carcinoma, radiotherapy, survival analysis

## Abstract

**Objective:**

Laryngeal verrucous carcinoma (LVC) comprises 1% to 4% of all laryngeal tumors. Although controversial, surgery has been the mainstay of treatment, due to concern about anaplastic transformation with radiotherapy. We aimed to study LVC patients to identify treatment patterns for primary and recurrent diseases.

**Study Design:**

Retrospective cohort study.

**Setting:**

Tertiary referral center.

**Methods:**

Patients with a pathological diagnosis of LVC treated over a 28‐year period were included. Baseline demographics, and treatment outcome measures including 5‐year laryngeal preservation rates (LPR), overall survival (OS), and recurrence‐free survival (RFS) were included. A literature review of published studies within the same study period was also completed.

**Results:**

Thirty‐two patients were included in the analysis (median age 61.5 years, 93.8% [30/32] male). Twenty‐three patients had T1 disease, and 9 had T2 disease with no evidence of regional or metastatic disease. The most common presenting symptom was hoarseness (93.8%) and the majority within the glottis 81.3% (26/32). Twenty‐nine patients underwent primary surgery only (28 local excisions, 1 vertical partial laryngectomy) meanwhile 3 underwent local excision with postoperative radiotherapy. LPR, OS, and RFS at 5 years were 95.8%, 90.1%, and 80.6%, respectively. Our literature review identified 23 previous studies, mostly single‐institution retrospective case series. Our study was the largest Canadian study in the literature to date.

**Conclusion:**

All LVC patients were treated with primary surgery, consistent with the current literature with excellent 5‐year OS and LPR. There was no consensus on the treatment of recurrent disease. Future prospective multicenter studies are warranted to further study this rare disease population.

Verrucous carcinoma, also known as Ackerman's tumor is a variant of squamous cell carcinoma (SCC) with low rates of metastasis.^1^ Associated risk factors include alcohol consumption, smoking, and infection with the oncogenic human papillomavirus (HPV) strains.^2^, ^3^, ^4^ The most common sites affected include the oral cavity (56%) and the larynx (35%).^5^ Within the larynx, laryngeal verrucous carcinoma (LVC) comprises 1% to 4% of all laryngeal tumors and most often affects middle‐aged men.^6^, ^7^, ^8^ The most common site of LVC is in the glottis (74.0%) followed by the supraglottis (9.2%) and then the subglottis.^2^ On gross examination, it has a fungating warty appearance and within the larynx, it has a propensity for glottic involvement.^9^, ^10^ The most common presenting symptom is hoarseness followed by dyspnea, dysphagia, and possibly upper airway obstruction.^2^, ^9^

Given its slow‐growing nature and rarity for regional or distant spread, there has been controversy in the treatment approach for this tumor. Traditionally, there has been hesitance in primary radiotherapy treatment due to concern for anaplastic transformation following this modality.^9^, ^11^ As a result, surgery has been employed most of the time. Surgical therapy most commonly includes local endoscopic excision followed less commonly by partial/hemilaryngectomy or total laryngectomy. Overall, surgery has shown better survival rates when compared to radiotherapy or a combination of radiotherapy and surgery.^2^

A best practice recommendation published by the Triological Society considered radiotherapy an acceptable treatment modality for LVC.^12^ However, surgery is still seen to achieve the highest locoregional control and demonstrates improved survival compared to radiotherapy.^12^ As this is a rare tumor, there has been a lack of prospective studies within this disease realm and thus, there still exists no definitive treatment strategy for the treatment of LVC.^13^ This study aimed to identify LVC patients treated in a Canadian tertiary care academic center serving the whole Canadian province of British Columbia over a 28‐year period and to identify treatment patterns for primary and recurrent diseases.

## Methods

Ethics Approval was obtained from the University of British Columbia Clinical Research Ethics Board (H18‐03134) to perform a retrospective cohort study. Adult patients (>18 years of age) who were treated with a pathological diagnosis of LVC between January 1, 1993, and January 1, 2021, were included in the review. Patients were identified through the Department of Pathology's Database with a keyword search for “verrucous,” “larynx,” “carcinoma,” “vocal cord,” or “vocal fold.” The pathologist then reviewed the pathology slides to ensure that the diagnosis of LVC was correct. A total of 32 charts were identified, reviewed, and included in the final analysis. The primary outcome was 5‐year overall survival (OS) and recurrence‐free survival (RFS). Secondary outcomes included local recurrence and 5‐year laryngeal preservation rates (LPR).

Baseline demographics information included age, gender, ethnicity, alcohol consumption (yes/no), smoking status (current/former/never smoker), and past medical history. On initial diagnosis, the patient's presenting symptoms, anatomical site of involvement (supraglottis/glottis/subglottis), and evidence of vocal fold impairment/fixation from endoscopy findings were recorded. The clinicopathologic characteristics of the neoplasm were recorded in accordance with the TNM staging (American Joint Committee on Cancer 8th edition).^14^ Treatment notes were reviewed to determine the primary treatment modality being one or a combination of surgery (local excision, partial/hemilaryngectomy, total laryngectomy), radiotherapy, or chemotherapy.

Details regarding the follow‐up number and length were compiled. Recurrence data was included with regard to the date and length of time from primary diagnosis. The treatment modality was confirmed with a review of the clinical notes and operative reports. This study followed the Strengthening the Reporting of Observational Studies in Epidemiology reporting guideline for cohort studies.^15^

### Statistical Analysis

Descriptive statistics were employed to analyze the baseline characteristics of the patients included in the retrospective review. Univariate analysis was performed to analyze the outcome measures with the inclusion of the median, and interquartile range (IQR). Survival analysis was performed to produce a Kaplan‐Meier curve measuring the 5‐year OS and RFS. All statistical analysis was performed using R statistical software (www.r-project.org).^16^ Due to the small sample size, multivariable regression was not performed to assess for the test of significance.

### Literature Review

A comprehensive literature review was performed to include all clinical studies pertaining to LVC. The search utilized the MEDLINE and Embase electronic bibliographical databases. The search strategy used the following terms: (1) “Larynx,” (2) “Exp Carcinoma, Squamous Cell/,” (3) “Laryngeal Neoplasms,” (4) “Verrucous Carcinoma.mp,” (5) “Laryngeal Verrucous Carcinoma.mp,” (6) “Carcinoma, Verrucous,” (7) “1 OR 2 OR 3,” (8) “4 OR 5 OR 6,” (9) “7 and 8,” (10) limit 9 to yr = “1993‐2021.” Extracted outcomes included a year of publication, country, sample size, treatment modality, survival outcomes, and level of evidence based on the Oxford Center for Evidence‐Based Medicine.^17^ If the articles did not include outcomes information of either treatment modality or survival data, they were excluded. Articles published between 1993 and 2021 were reviewed.

## Results

Thirty‐two patients were included in the final analysis. The median age of the patient cohort was 61.5 years (IQR: 20.5). LVC had a predilection for males (30/32) with a ratio of 15:1. Most of the patients were of Caucasian ethnicity (78.1%). With regards to smoking status, 21.9%, 40.6%, and 37.5% of patients were never, current, and former cigarette smokers, respectively. Twenty patients endorsed current alcohol consumption although the content and frequency were not specified. For patients with follow‐up information, the median duration of patient follow‐up was 22 months. The demographics of the patient population are summarized in Table 1.

**Table 1:** Patient Demographics, Clinical Symptoms, and Anatomic Location of Laryngeal Verrucous Carcinoma

Age (y)	
Range	39‐81
Interquartile range	20.5
Median	61.5
Race, N, %	
Caucasian	25 (78.1)
Asian	4 (12.5)
Not specified	3 (9.4)
Gender, N	
Male	30
Female	2
Smoking, N, %	
Never	7 (21.9)
Current	13 (40.6)
Former	12 (37.5)
Alcohol consumption, N, %	
Yes	20 (62.5)
No	12 (37.5)
Presenting symptoms, N, %	
Hoarseness	30 (93.8)
Shortness of breath	1 (3.1)
Sore throat	1 (3.1)
Anatomic site, N, %	
Glottis	26 (81.3)
Supraglottis	0 (0)
Subglottis	0 (0)
Extension to subglottis/supraglottis	6 (18.7)

The most common presenting symptom was hoarseness (93.8%). Other symptoms included shortness of breath or sore throat. The anatomical subsite of primary tumor origin was glottic in all patients. Six patients had extensions into the subglottic or supraglottic region from their primary glottic site. Twenty‐three patients (71.9%) had T1, and 9 (28.1%) had T2 disease from the clinical examination (Table 2). None of the patients had evidence of vocal fold impairment or immobility. There was no regional or distant metastasis within the clinical cohort. Following a review of the pathology report by the pathologist, 100% of the patients had confirmation of verrucous SCC on their specimen slides.

**Table 2:** TNM Staging for Primary Laryngeal Verrucous Carcinoma Patients

TNM staging	N (%)
T1	23 (71.9)
T2	9 (28.1)
T3	0 (0)
T4	0 (0)
N0	0 (0)
N1	0 (0)
N2	0 (0)
N3	0 (0)
NX	0 (0)
M0	0 (0)
MX	0 (0)

The treatment modality of the primary malignancy included surgery alone or surgery with postoperative radiotherapy (Table 3). Twenty‐nine patients (90.6%) underwent primary surgery with 28 having a local excision via transoral laser microsurgery (TLM) and 1 having a vertical partial laryngectomy given the extent of disease as it was not amenable to TLM. Meanwhile, 3 patients (9.4%) underwent local excision via TLM followed by postoperative radiotherapy. No patients underwent postoperative chemotherapy. No patients were treated with sole radiotherapy for the treatment of primary disease. In the primary surgical group, 26 and 3 patients had T1 and T2 diseases, respectively. Within the surgery and postoperative radiotherapy group, 1 patient had T1 disease while 2 had T2 disease.

**Table 3:** Treatment of Primary Disease, Recurrence Incidence, Treatment of Recurrent Disease, and Overall Survival Outcomes

Primary disease treatment	N (%)
Surgery (local excision)	28 (87.5)
Surgery (vertical partial laryngectomy)	1 (3.1)
Surgery and radiotherapy	3 (9.4)
Recurrence	
Yes	14 (43.8)
No	18 (56.2)
Recurrent disease treatment	14
Surgery	10 (71.4)
Radiotherapy	2 (14.3)
Surgery and radiotherapy	2 (14.3)

Clinical information including demographics, presenting symptoms, primary treatment, and salvage therapy can be referenced in Table 4. Local recurrence occurred in 43.8% (14/32) of the primary surgical group patients (9 in T1, 5 in T2). In the recurrence group, 10 patients had surgical therapy, 2 had radiotherapy, and 2 patients had surgery with postoperative radiotherapy. Within the subset that underwent repeat surgical therapy for recurrence, 1 patient had a supraglottic laryngectomy while the remaining patients underwent local excision via TLM. None of the 3 patients who had combined modality (surgery with radiotherapy) for their primary disease had a recurrence on their latest follow‐up visit. Two patients with prior LVC treatment eventually had a diagnosis of invasive SCC on their latest biopsy for which 1 underwent salvage radiotherapy and the other had a total laryngectomy as they had radiotherapy 3 years prior to the development of SCC. Nevertheless, the LPR at 5 years was 95.8%.

**Table 4:** Clinical Characteristics and Treatments in the 32 Patients With Laryngeal Verrucous Carcinoma

ID	Age	Sex	T‐stage	Site	Overall stage	Symptom	Primary surgery	Adjuvant XRT	Recurrence	Salvage therapy
1	64	M	1	G	1	H	TLM	−	−	−
2	75	M	2	G	2	H + S	TLM	+	−	−
3	77	F	2	G	2	H	TLM	−	+	XRT
4	50	M	1b	G	1	H	TLM	+	−	−
5	81	M	1a	G	1	H	TLM	−	+	Sx
6	39	M	1a	G	1	H	TLM	−	−	−
7	58	M	2	G	1	H	TLM	−	+	Sx
8	47	M	1a	G	1	H	TLM	−	−	−
9	78	M	1a	G	1	H	TLM	−	+	Sx
10	70	M	2	G + Sub	2	H	TLM	+	−	−
11	45	M	1a	G	1	H	TLM	−	+	Sx + XRT
12	58	M	1b	G	1	H	TLM	−	+	Sx + XRT
13	48	M	1a	G + Sub	1	S	TLM	−	−	−
14	75	M	1a	G	1	H	TLM	−	−	−
15	66	M	1b	G	1	H	TLM	−	+	Sx
16	62	M	1b	G	1	H	TLM	−	−	−
17	54	M	1a	G	1	H	TLM	−	+	Sx
18	63	M	2	G + Sub	1	H	TLM	−	+	Sx
19	47	M	2	G	2	H	VPL	−	−	−
20	61	M	2	G + Supra	2	H	TLM	−	+	Sx
21	72	M	2	G + Supra/Sub	1	H	TLM	−	−	−
22	66	M	1a	G	1	H	TLM	−	−	−
23	56	M	2	G + Sub	1	H	TLM	−	+	Sx
24	72	M	1a	G	1	H	TLM	−	−	−
25	41	M	1a	G	1	ST	TLM	−	−	−
26	43	M	1a	G	1	H	TLM	−	+	Sx
27	53	M	1b	G	1	H	TLM	−	−	−
28	62	M	1a	G	1	H	TLM	−	−	−
29	73	M	1a	G	1	H	TLM	−	−	−
30	52	F	1a	G	1	H	TLM	−	+	XRT
31	58	M	1a	G	1	H	TLM	−	−	−
32	75	M	1b	G	1	H	TLM	−	+	Sx

Abbreviations: F, female; G, glottis; H, hoarseness; M, male; S, shortness of breath; ST, sore throat; Sub, subglottis; Supra, supraglottis; Sx, surgery; TLM, transoral laser microsurgery; XRT, radiotherapy.

**Figure 1:**
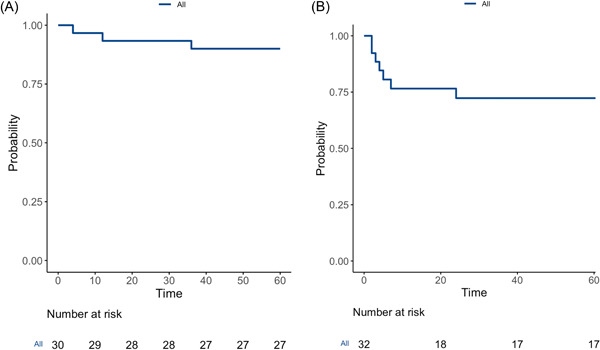
(A) Five‐year overall survival for all patients in the study cohort. (B) Five‐year recurrence‐free survival for all patients in the study cohort.

Five‐year OS and RFS were 90.1% and 80.6%. At 5 years, 3 patients had died, of which 1 was dead from other causes, and 2 were not otherwise specified due to the lack of information available in the medical records. As patients were lost to follow‐up at different time intervals following the intervention, a Kaplan‐Meier curve was deemed to be appropriate to demonstrate the 5‐year OS and RFS as shown in Figure 1A and B.^18^

### Literature Review

Table 5 shows a summary of the published literature on LVC between 1993 and 2021. Overall, 23 studies were identified through the literature review. Three studies were excluded because they did not include any survival or treatment‐related data as they focused on other aspects of LVC (eg, pathology, radiology). The levels of evidence of the included studies were level 5 (n = 10) and level 4 (n = 13). The country with the most published studies included the United States of America. The number of patients ranged from 1 to 53. The total number of patients was 305 of which 259 had primary surgery, 30 primary radiotherapy, 14 surgery with postoperative radiotherapy, 1 photodynamic therapy, and 1 oral chemotherapy. Although most patients underwent local excision, other procedures included partial or total laryngectomy, supraglottic laryngectomy, and laryngofissure. Within the primary surgery group (n = 259), 148 underwent a local excision whereas 111 underwent an open procedure. Within the surgery and postoperative radiotherapy group, 5 and 9 patients underwent local excision and an open procedure, respectively. The specific open procedures with respect to each study can be seen in Table 5.

**Table 5:** Review of Published Literature on Laryngeal Verrucous Carcinoma

Author	Year	Country	n	Study design	Treatment modality (primary disease)	Survival data	CEBM
Sagit et al^19^	2016	Turkey	1	Case report	*Sx* 1—LE	ANED at 1 y	5
Staltari et al^20^	2015	USA	1	Case report	*Sx* 1—LE	ANED at 7 mo	5
Triaridis et al^21^	2014	Greece	1	Case report	*Sx* 1—LE + TL + ND	ANED at 3 y	5
Cuny et al^22^	2013	France	2 of 59	Case series	*RT* (2)	N/A	4
Hod et al^3^	2010	Israel	18	Case series	*Sx* (13) 13—LE *SRT* (5) 4—LE 1—LE + Laryngofissure	100% Survival at 3 mo 13—ANED 5—AWD requiring further surgery or RT	4
Witt et al^23^	2009	USA	1	Case report	*SRT* 1—TL	ANED at 18 mo	5
Karahatay et al^24^	2007	Turkey	1	Case report	*Sx* 1—TL + ND	ANED at 2 y	5
Strojan et al^4^	2006	Slovenia	30	Case series	*Sx* (23) 3—LE 5—Thyrofissure 5—PL 10—TL *RT* (7)	*5 y* Local FFS 97% DSS 100% OS 75% *7.5 y* 14 ANED 16 DOC	4
Motta et al^25^	2005	Italy	46 of 719	Case series	*Sx* 46— LE	N/A	4
Kapur et al^26^	2005	USA	1	Case report	*Sx* 1—TL + ND	N/A	5
Varshney et al^6^	2004	India	1	Case report	*Sx* 1—Laryngofissure	ANED at 2 mo	5
Shvero et al^27^	2003	Israel	3 of 26	Case series	*Sx* 3—LE	3—ANED between 24‐32 mo	4
Remijn et al^28^	2002	Netherlands	5 of 43	Case series	*Sx* 5—LE	4—ANED 1—AWD (underwent repeat surgical excision)	4
McCaffrey et al^29^	1998	USA	52	Case series	*Sx* (52) 16—LE 20—PL 12—TL 4—SGL	5‐y recurrence‐free survival 71%	4
Orvidas et al^30^	1998	USA	53	Case series	*Sx* (49) 20—LE 16— PL 8—TL 3—SGL 2—LP *RT* (3) *SRT* (1) 1—LP	3‐y DFS—82.7% *Overall survival* 78% at 5 y 59% at 10 y	4
Damm et al^31^	1997	Germany	21	Case series	*Sx* (21) 21—LE	19—ANED 6‐122 mo 2—DOC	4
Kawaida et al^32^	1997	Japan	1	Case report	*Sx* 1—LE	ANED at 21 mo	5
Maurizi et al^33^	1996	Italy	31	Case series	*Sx* (23) 8—LE 4—LS 1—HL 2—FLL 4—TL 3—TL + ND 1—HL + ND *SRT* (7) 1—LS 2—TL 1—TL + ND 1—SGL + ND 1—HL 1—FLL *No treatment* (1)	*Sx*: 1 DOC *SRT*: 2 DOD	4
Abdulla and Hasselt^34^	1995	Hong Kong	1	Case report	*Sx* 1—LE	ANED at 3 y	5
Biel^35^	1994	USA	1 of 11	Case series	1—PDT	ANED at 27 mo	4
Fliss et al^36^	1994	Canada	22 of 29	Case series	*Sx* (6) 5—HL 1—TL *RT* (16)	*Sx*: 5—ANED 6‐33 mo 1—DOC *RT*: 10—ANED 8‐122 mo 6—AWD 4‐130 mo	4
Kitano and Kitajima^37^	1994	Japan	1	Case report	Tegafur (chemo)	ANED at 30 mo	5
Hagen et al^9^	1993	USA	12	Case series	*Sx* (10) 2—laryngofissure 5—LE 3—TL *RT* (2)	83% DFS at 3‐12 y	4

Abbreviations: ANED, alive with no evidence of disease; AWD, alive with disease; CEBM, Center for Evidence‐Based Medicine; DFS, disease‐free survival; DOC, dead of other cause; DOD, dead of disease; FFS, failure‐free survival; FLL, frontolateral laryngectomy; HL, hemilaryngectomy; LE, local excision; LP, laryngopharyngectomy; LS, local stripping; NA, not available; ND, neck dissection; NOS, not otherwise specified; OS, overall survival; PDT, photodynamic therapy; PL, partial laryngectomy; RT, radiotherapy; SGL, supraglottic laryngectomy; SRT, surgery + postoperative radiotherapy; Sx, surgery; TL, total laryngectomy.

## Discussion

LVC is a locally invasive variant of SCC with a rarity for regional or distant spread.^1^ In this retrospective cohort study over a 28‐year period, we assessed 32 adult patients from a tertiary care academic center that services the whole Canadian province of British Columbia diagnosed with LVC. The average cohort was middle‐aged Caucasian men which is consistent with the epidemiology cited in the literature.^2^ In this study, all patients presented with the local disease at stage T1/T2 where the majority of patients had disease confined to the glottis. 18.7% of patients had an extension to the subglottis/supraglottis. Furthermore, nearly all patients (93.8%) presented with hoarseness which is not surprising given LVC's propensity for the glottis as opposed to other subsites of the larynx.^7^

Traditionally, there has been controversy in the treatment of LVC with either surgery or radiotherapy. One hesitation toward the treatment of LVC with radiotherapy has been due to concern for anaplastic transformation.^9^, ^11^ Within our cohort, 1 patient with 3 prior local excisions undergoing radiotherapy for their third recurrence developed an SCC. Their SCC developed 3 years following radiotherapy for which they underwent a total laryngectomy. Another patient had a spontaneous SCC transformation, but they had not previously received radiotherapy. Our cohort results also demonstrate that the risk of anaplastic transformation is negligible, and the benefits of treatment far outweigh the risk of developing SCC especially when a patient is not a surgical candidate.^11^, ^12^

Even though the literature demonstrates controversy in the treatment of primary LVC, this was not the case within our institutional cohort.^2^, ^7^ All our patients underwent surgery as their primary surgical therapy, of which 3 out of 32 (9.4%) had additional postoperative radiotherapy. Recurrence occurred in 43.8% of patients which is higher than the rates reported in the literature.^2^ In a systematic review by Echanique et al looking at a total of 369 patients, 81.2% did not have a recurrence and 11.7% of patients had evidence of local recurrence.^2^ However, all studies were either case series or case reports and may have had different standards for tumor surveillance. Although the recurrence rate was higher in our cohort, most patients required 1 additional surgery or salvage radiotherapy to be disease‐free. Additionally, there was no disease‐specific mortality which is consistent with the diagnosis of LVC. Finally, there were excellent 5‐year OS, RFS, and LPRs. Nevertheless, the higher recurrence rate represents an important finding that requires future attention in identifying factors that predispose patients to develop recurrent LVC.

Although surgery has resulted in better OS and disease‐free survival, radiotherapy should not be discounted altogether.^2^ To date, there have been no controlled prospective studies to directly compare the 2 treatment modalities. Additionally, patients may have surgical contraindications or multiple comorbidities which preclude them from being surgical candidates. There may have also been a selection bias in these retrospective reviews (without internal controls) as patients with more severe disease or more recurrent diseases were treated with adjuvant radiotherapy. Therefore, we believe that radiotherapy is still an acceptable alternative should a patient not be a surgical candidate. In fact, a recent publication by the Triological Society stated that radiotherapy is an acceptable alternative for the treatment of LVC as the concern for anaplastic transformation is negligible and radiotherapy treatment for other non‐verrucous pathologies has shown good functional results.^12^

Although we identified 23 studies in the literature review, only 9 studies had a cohort size greater than 10 patients. Our study adds to the literature by being the largest study published since 2010. Specifically, this is only 1 of 2 Canadian studies pertaining to the treatment of LVC. The other Canadian study was published in 1994 and had a smaller sample size of 22.^36^ Moreover, its primary objective was to determine the prevalence and HPV typing within LVC patients.^36^ Therefore, this is the first Canadian study aimed at studying the treatment and survival outcomes in patients with LVC. Our study reports results from a universal public system, where barriers to insurance and lower socioeconomic status may be less pronounced. This difference is especially important in head and neck cancer patients as they are usually from lower socioeconomic status.^38^ Our patients were all treated with primary surgery which is consistent with the findings within the review of most patients who were treated with primary surgery. In the literature review for the primary surgery group (n = 259), 148 underwent a local excision whereas 111 underwent an open procedure. However, our results would show that patients undergoing local excision via TLM can still have excellent LPRs and survival without requiring an open procedure. We do however acknowledge the multifactorial nature of this rare pathology and therefore, further prospective studies are required to determine the optimal surgical modality for patients presenting with LVC. Information such as margin status was not frequently reported within the reports; some patients may have initially undergone a biopsy as an initial procedure and definitive management thereafter for total removal. Therefore, this may have accounted for the higher recurrence seen within our cohort. However, no patients died from their disease and most patients only required 1 additional surgery or radiotherapy.

Several limitations do exist within this study. Given its retrospective nature, follow‐up information was not available for all patients. Some patients were lost to follow up which placed a constraint on analyzing the patient's survival and rate of recurrence. Overall, the diagnosis of LVC has been challenging in the past as it can be misclassified as benign or traditional SCC.^30^ It can also be difficult to identify a hybrid SCC which can increase a patient's risk of recurrence or potential for spread.^30^ This is a limitation present within any study pertaining to LVC. However, to ensure that we included only LVC cases, the pathologist reviewed each patient's slides to confirm that the diagnosis of LVC was correct. Furthermore, our study is strengthened by the inclusion of a literature review that summarizes publications that assess the treatment modality of LVC and associated outcomes in the past 28 years. Within the review, most patients underwent surgical treatment, and the rate of anaplastic transformation was quite rare which was consistent with our findings. Therefore, the role of radiotherapy should be further studied as it can be considered in patients who are not optimal surgical candidates. Within the literature review, the level of evidence was levels 4 to 5. Given the rarity of this disease, prospective multi‐institutional studies are needed to better understand the disease evolution to develop criteria that will delineate which patients may benefit from a surgical or radiotherapy treatment approach.

## Conclusions

In this cohort, all LVC patients were treated with primary surgery, 3 of which also underwent postoperative radiotherapy. The rate of recurrence was 43.8%; although higher was controlled with further surgery or radiotherapy. Additionally, our patient had excellent laryngeal preservation even with additional surgical interventions. Although the treatment of primary LVC is less controversial, the treatment of recurrent disease with either surgery or radiotherapy is not well defined. Future multi‐institutional prospective studies are therefore warranted to directly compare the 2 treatment modalities for this disease population.

## Authors Contributions

**Ameen Amanian**, was a part of drafting the research protocol, data collection, data analysis, preparation of the manuscript, and revisions of the final manuscript; **Donald W. Anderson**, **James Scott Durham**, **Eitan Prisman**, **Tony Ng**, contributed to the study conception and design, revision, and final approval of the manuscript; **Amanda Hu**, conceived the project and research protocol and revised the manuscript critically for important intellectual content.

## Disclosures

### Competing interests

The author(s) declared no potential conflicts of interest with respect to the research, authorship, and/or publication of this article.

### Funding source

None.
